# Inhibition of Hyaluronic Acid Synthesis Suppresses Angiogenesis in Developing Endometriotic Lesions

**DOI:** 10.1371/journal.pone.0152302

**Published:** 2016-03-28

**Authors:** Carla N. Olivares, Laura D. Alaniz, Michael D. Menger, Rosa I. Barañao, Matthias W. Laschke, Gabriela F. Meresman

**Affiliations:** 1 Instituto de Biología y Medicina Experimental (IBYME-CONICET), Ciudad Autónoma de Buenos Aires, Argentina; 2 CIT NOBA, Universidad Nacional del Noroeste de la Provincia de Buenos Aires (CONICET-UNNOBA), Junín, Buenos Aires, Argentina; 3 Institute for Clinical & Experimental Surgery, University of Saarland, Homburg/Saar, Germany; CHU Clermont-Ferrand, FRANCE

## Abstract

**Background:**

The development and long-term survival of endometriotic lesions is crucially dependent on an adequate vascularization. Hyaluronic acid (HA) through its receptor CD44 has been described to be involved in the process of angiogenesis.

**Objective:**

To study the effect of HA synthesis inhibition using non-toxic doses of 4-methylumbelliferone (4-MU) on endometriosis-related angiogenesis.

**Materials and Methods:**

The cytotoxicity of different *in vitro* doses of 4-MU on endothelial cells was firstly tested by means of a lactate dehydrogenase assay. The anti-angiogenic action of non-cytotoxic doses of 4-MU was then assessed by a rat aortic ring assay. In addition, endometriotic lesions were induced in dorsal skinfold chambers of female BALB/c mice, which were daily treated with an intraperitoneal injection of 0.9% NaCl (vehicle group; n = 6), 20mg/kg 4-MU (n = 8) or 80mg/kg 4-MU (n = 7) throughout an observation period of 14 days. The effect of 4-MU on their vascularization, survival and growth were studied by intravital fluorescence microscopy, histology and immunohistochemistry.

**Main Results:**

Non-cytotoxic doses of 4-MU effectively inhibited vascular sprout formation in the rat aortic ring assay. Endometriotic lesions in dorsal skinfold chambers of 4-MU-treated mice dose-dependently exhibited a significantly smaller vascularized area and lower functional microvessel density when compared to vehicle-treated controls. Histological analyses revealed a downregulation of HA expression in 4-MU-treated lesions. This was associated with a reduced density of CD31-positive microvessels within the lesions. In contrast, numbers of PCNA-positive proliferating and cleaved caspase-3-positive apoptotic cells did not differ between 4-MU-treated and control lesions.

**Conclusions:**

The present study demonstrates for the first time that targeting the synthesis of HA suppresses angiogenesis in developing endometriotic lesions. Further studies have to clarify now whether in the future this anti-angiogenic effect can be used beneficially for the treatment of endometriosis.

## Introduction

Endometriosis is a benign gynecological disease, which is characterized by endometrial foci outside the uterus. It is estimated that it affects up to 10% of women of reproductive age. In infertile women the prevalence rate is even higher [1]. Apart from infertility, endometriosis patients often experience severe abdominal pain during menses and in occasions along the cycle, markedly affecting their everyday life. Since Sampson’s postulation of the retrograde menstruation theory, numerous studies have been conducted to elucidate the pathogenic mechanisms, which contribute to the development of the disease and to identify novel therapeutic targets [2,3].

Current, conventional pharmacological treatment of endometriosis focuses on the generation of a hypoestrogenic environment, achieving it by modulation of steroid hormones through oral contraceptives, progestins and GnRH analogues. The rationale behind this, is that the onset and growth of endometriotic lesions is crucially dependent on estrogen stimulation [4]. However, this may be associated with the typical side effects of an anti-estrogenic therapy, such as hot flashes, episodes of depression and osteoporosis [5]. Accordingly, alternative approaches are currently under investigation. These include the treatment of active endometriotic lesions with anti-angiogenic compounds or the suppression of endometriosis-associated adhesion and tissue invasion [6,7].

CD44 receptor and hyaluronic acid or hyaluronan (HA) have been identified as key molecules involved in the adhesion of menstrual endometrial cells to peritoneal cells [8,9]. Griffith *et al*. found that endometrial cells from women with endometriosis exhibit a high expression of several CD44 isoforms, which increases their rate of adherence to peritoneal mesothelial cells when compared to endometrial cells of controls [9]. Hasegawa et al. [10] further suggested that the relative proportion of CD44 and its soluble form sCD44 in peritoneal fluid is important for the establishment and progression of endometriosis.

The principal ligand for CD44 is HA [11]. HA is a linear, non-sulphated glycosaminoglycan and one of the main components of the extracellular matrix. Its synthesis is dependent on three enzymes: hyaluronan synthase (HAS)-1, HAS-2 and HAS-3, whereas several hyaluronidases degrade HA into fragments of different sizes [12]. These fragments have been shown to be potent stimulators of angiogenesis [13,14]. Of interest, HA is detectable in normal, hyperplastic and neoplastic endometrium [15,16]. However, the site of HA production in endometriosis is unknown so far. Nonetheless, the findings of Hasegawa et al. [17] indicate an important role of HA in the regulation of endometriotic lesion formation. They found that administration of a HA solution in the peritoneal cavity of mice significantly suppresses the engraftment of inoculated endometrial fragments by preventing the interaction of CD44-positive endometrial cells with the peritoneum.

The synthesis of HA can be suppressed by 4-methylumbelliferone (4-MU), which depletes cells from one of the substrates necessary for its synthesis, i.e. UDP-glucuronic acid, and downregulates the transcription of two of the synthases [18]. 4-MU is a coumarin of vegetal origin which originates from umbelliferous plants. It has originally been described as a choleretic and spasmolytic agent and has been tested in a phase II clinical trial for hepatitis B and C patients without inducing severe side effects ([19,20] and ClinicalTrials.gov identifier: NCT00225537), which indicates that 4-MU is well tolerated. Of interest, 4-MU has been shown in vitro and in vivo to be an effective inhibitor of cell proliferation, migration, angiogenesis, tumor growth and metastasis [21–24].

Given the background on HA and 4-MU, and the potential involvement of CD44-HA interaction in the pathogenesis of endometriosis, the aim of the present study was to analyze for the first time the effects of 4-MU on endometriosis-related angiogenesis. With both in vivo and in vitro approaches we demonstrate that inhibiting the synthesis of HA reduces vascularization and microvessel density.

## Materials and Methods

### Lactate dehydrogenase (LDH) assay

To assess the cytotoxicity of different doses of 4-MU on endothelial cells, we performed a LDH assay by means of the Cytotoxicity Detection KitPLUS (Roche, Mannheim, Germany) according to the manufacturer's instructions. For this purpose, human dermal microvascular endothelial cells (HDMEC; PromoCell, Heidelberg, Germany) were cultured in Endothelial Cell Growth Medium MV (PromoCell) at 37°C under a humidified 95% to 5% (v/v) mixture of air and CO_2_ until they reached confluence. The cells were then stimulated with 1, 2 and 4mM 4-MU (n = 4 each) or 0.9% NaCl as vehicle control (n = 4). After 24h, 100μL of reaction mix per 100μL medium was added to each well. After 10min at room temperature in the dark, the reaction was stopped by addition of 50μL stop solution. Absorption was then measured at 492nm with 620nm as reference using a microplate reader and corrected to blank values (wells without cells). As a positive control cells were treated with a lysis buffer and subjected to the same procedure. The concentrations tested were chosen according to previous studies [21,25].

### Animals

All animal care and experimental procedures were approved by the local governmental animal care committee (Landesamt für Verbraucherschutz, Saarbrücken, Germany; Permit Number: 53/2011) and were conducted in accordance with the European legislation on protection of animals (Guide line 2010/63/EU) and the NIH Guidelines for the Care and Use of Laboratory Animals (http://oacu.od.nih.gov/regs/index.htm. 8th Edition; 2011).

Adult male Sprague Dawley rats with an average weight of 270g were used for the aortic ring assay. For the endometriosis study, 3–4 months old female BALB/c mice with an average weight of 24g were used. The animals were kept individually caged in a 12h light/dark cycle with free access to water and standard pellet food (Altromin, Lage, Germany). To exclude discrepancies between individual animals due to differences in steroid hormone synthesis, only animals in the stage of estrous were used as donors and recipients for the transplantation of endometrial fragments into the dorsal skinfold chamber. For this purpose, the cycle stage was determined by vaginal cytology [26].

### Aortic ring assay

Aortic rings from 4 adult male Sprague Dawley rats were embedded in 200μL Matrigel (BD Matrigel^™^ Matrix; BD Biosciences, Heidelberg, Germany) in 48-well tissue culture grade plates and allowed to polymerize for 20min at 37°C in a humidified atmosphere containing 5% CO_2_. Subsequently, the wells were overlaid with 800μL Dulbecco’s modified Eagle medium enriched with 10% fetal calf serum, 100U/mL penicillin and 0.1mg/mL streptomycin (complete medium, PAA, Cölbe, Germany), which was supplemented with 0.9% NaCl (vehicle), 1, 2 or 4mM 4-MU. The rings were cultured for 6 days with medium change at day 3. Each experimental group consisted of 10 rings. Vascular sprouting from each ring was examined by a BZ-8000 microscope (Keyence, Osaka, Japan) and analyzed by means of the software package CapImage (version 8.5; Dr. Zeintl, Heidelberg, Germany). The quantitative analyses included the determination of the sprout area (mm^2^), i.e. the overall area of the outer aortic vessel sprouting, and the maximal sprout length (μm), i.e. the longest perpendicular distance from the aortic ring to the outer border of vascular sprouting.

### Preparation of the dorsal skinfold chamber

The dorsal skinfold chamber model has already been described in detail [27–29]. Briefly, animals were anesthetized by intraperitoneal (i.p.) injection of ketamine (75mg/kg body weight; Ursotamin^®^, Serumwerk Bernburg, Bernburg, Germany) and xylazine (15mg/kg body weight; Rompun^®^, Bayer, Leverkusen, Germany). After removal of the dorsal hair, two symmetrical titanium frames were implanted on the extended dorsal skinfold of the animals, so that they sandwiched the double layer of skin. One layer of skin was completely removed in a circular area of approximately 15mm in diameter. The remaining layers consisting of striated skin muscle, subcutaneous tissue and skin were covered with a removable cover glass, which was fixed in one of the titanium frames by means of a snap ring. After the preparation, the animals were allowed to recover from anesthesia and surgery for 48h. Body weight and behavior were monitored daily.

### Isolation of endometrial tissue fragments

Anesthetized mice served as donors of both uterine horns, which were transferred to a Petri dish containing complete medium. Because we demonstrated in a recent study [30] that the natural barrier function of the luminal epithelium prevents the interaction of endometrial grafts with the surrounding host tissue, we generated in the present study endometrial fragments without luminal epithelium. As previously described [30], uterine horns were opened longitudinally, myometrium was removed and fragments from the basal endometrium were excised and transferred to a Petri dish containing fresh warmed complete medium. Only fragments measuring 0.7mm in diameter were used for transplantation. All procedures were performed using a stereo microscope (M651; Leica Microsystems, Wetzlar, Germany).

### Transplantation of endometrial tissue fragments

Selected fragments were transferred into the dorsal skinfold chamber of recipient animals. For this purpose, the cover glass of the dorsal skinfold chamber was removed and the underlying tissue was flushed several times with 0.9% NaCl. Two fragments were placed onto the host striated muscle tissue within each chamber, with a maximal distance to each other to exclude their mutual interaction during the engraftment process. Finally, the chamber was closed again with a new cover glass. Before transplantation, fragments were incubated for 1min in complete medium with 200μg/mL of the fluorescent dye bisbenzimide (Sigma-Aldrich, Munich, Germany) and washed again in bisbenzimide-free medium. After transplantation, bisbenzimide allows for an easy differentiation of the stained endometrium from the surrounding non-stained host tissue [27].

From the day of endometrial tissue transplantation (d0) until the day of sacrifice (d14) the recipient animals were daily treated with 0.9% NaCl (vehicle group; n = 6), 20mg/kg 4-MU (n = 8) or 80mg/kg 4-MU (n = 7). 4-MU was injected intraperitoneally (i.p.). This route of administration has previously been shown to induce significant in vivo effects in a comparable dose range [24].

### Intravital fluorescence microscopy and image analysis

Vascularization of the developing endometriotic lesions in the dorsal skinfold chamber was assessed by means of intravital fluorescence microscopy, as described previously in detail [31]. For this purpose animals were anesthetized and injected i.v. with 5% of fluorescein isothiocyanate (FITC)-labeled dextran 150,000 (Sigma-Aldrich) for visualization of vessels. Intravital fluorescence microscopy was performed directly after endometrium transplantation as well as at days 3, 6, 10 and 14. The microscopic images were analyzed quantitatively offline by means of the software package CapImage (Dr. Zeintl). The analyses included the determination of the grafts’ size, the size of the vascularized area, i.e. the lesion area covered with microvessels in percentage of the overall area of the grafts, and the functional microvessel density, i.e. the length of red blood cell (RBC)-perfused microvessels per observation area (cm/cm^2^). At the end of the in vivo experiments (d14), the animals were sacrificed with an overdose of the anesthetics and the dorsal skinfold chamber preparations were further processed for histology and immunohistochemistry.

### Histology and immunohistochemistry

The effect of 4-MU on HA synthesis, cell proliferation (proliferating cell nuclear antigen, PCNA), apoptosis (cleaved caspase-3) and blood vessel formation (endothelial cell marker CD31) within endometriotic lesions was analyzed at day 14 by means of histology and immunohistochemistry. At sacrifice the dorsal skinfold chamber tissue was obtained and formalin fixed for subsequent paraffin embedding. Sections of 2μm thickness were cut and stained with hematoxilin and eosin (HE) and examined for the presence of endometriotic glands and stroma.

To evidence if the treatment with 4-MU effectively reduced the synthesis of HA within the endometriotic lesions, a HA staining was performed as described elsewhere [24]. Briefly, sections were incubated with 3% H_2_O_2_-methanol for 30min at room temperature (RT) to block endogenous peroxidase, followed by avidin, biotin and protein-blocking solution (VectorLabs, Burlingame, CA). Then, 5μg/mL of biotinylated HA binding protein (bHA-BP) (385911, Calbiochem, Billerica, MA, USA) diluted in 1% BSA-PBS was applied for 1h. Negative controls were stained with bHA-BP after treatment with 100U/mL of Streptomyces hyaluronidase (385931, Calbiochem) at 37°C for 30min. Peroxidase complex (Sigma-Aldrich) 1:10 in PBS was used as a revealing system. The signal was detected by 0.1% 3,3′-diaminobenzidine (DAB, Sigma-Aldrich), 4% glucose, 0.08% ammoniun chloride, 5% nickel ammonium sulfate in 0.2M sodium acetate and 0.05% hydrogen peroxide.

Furthermore, sections were subjected to standard immunohistochemistry procedures for the detection of PCNA and cleaved caspase-3. For the detection of CD31, immunofluorescence was performed. The primary antibodies used were: monoclonal mouse anti-PCNA (1:200; Dako Deutschland, Hamburg, Germany), polyclonal rabbit anti-cleaved caspase-3 (1:100; Cell Signaling Technology, Boston, MA, USA) and monoclonal rat anti-CD31 (1:30; Dianova, Hamburg, Germany). The corresponding secondary antibodies used were: biotin-labeled goat anti-mouse antibody (1:100; Abcam, Cambridge, UK), biotin-labeled goat anti-rabbit (ready-to-use; Abcam) and goat anti-rat Cy3 antibody (1:50; Dianova). For light microscopy, sections were further incubated with avidin-peroxidase (1:50; Sigma-Aldrich) and DAB. Nuclei staining was achieved with hematoxilin or Hoechst 33342 (1:500; Sigma-Aldrich), respectively.

HA-stained sections were analyzed under a standard light microscope (BX60; Olympus, Hamburg, Germany) and a semiquantitative score was assigned to each section with 0: no staining, 1: low staining, 2: mild staining and 3: intense staining.

The fraction of PCNA- and cleaved caspase-3-positive epithelial and stromal cells within each lesion was quantitatively assessed by light microscopy (BX60). For this purpose, all positive cells were counted and expressed as percentage of the total cell count per lesion. The density of CD31-positive microvessels was assessed on additional fluorescent sections using a BZ-8000 microscopic system with an image analysis software from Keyence (Osaka, Japan), which allowed the planimetric measurement of the lesion area on each section. The counted number of microvessels per lesion was then divided by this area to express the microvessel density as mm^-2^.

### Statistics

Data were first analyzed for normal distribution and equal variance. One way ANOVA or two ways repeated measures ANOVA were performed, according to experimental design, followed by the Student Newman-Keuls test (SigmaStat; Jandel Corporation, San Rafael, CA). All values are expressed as means ± SEM. Correlation index was calculated using the Pearson Product Moment Correlation (SigmaStat). Statistical significance was accepted for a value of p<0.05.

## Results

### Cytotoxicity of 4-MU

**Fig 1:**
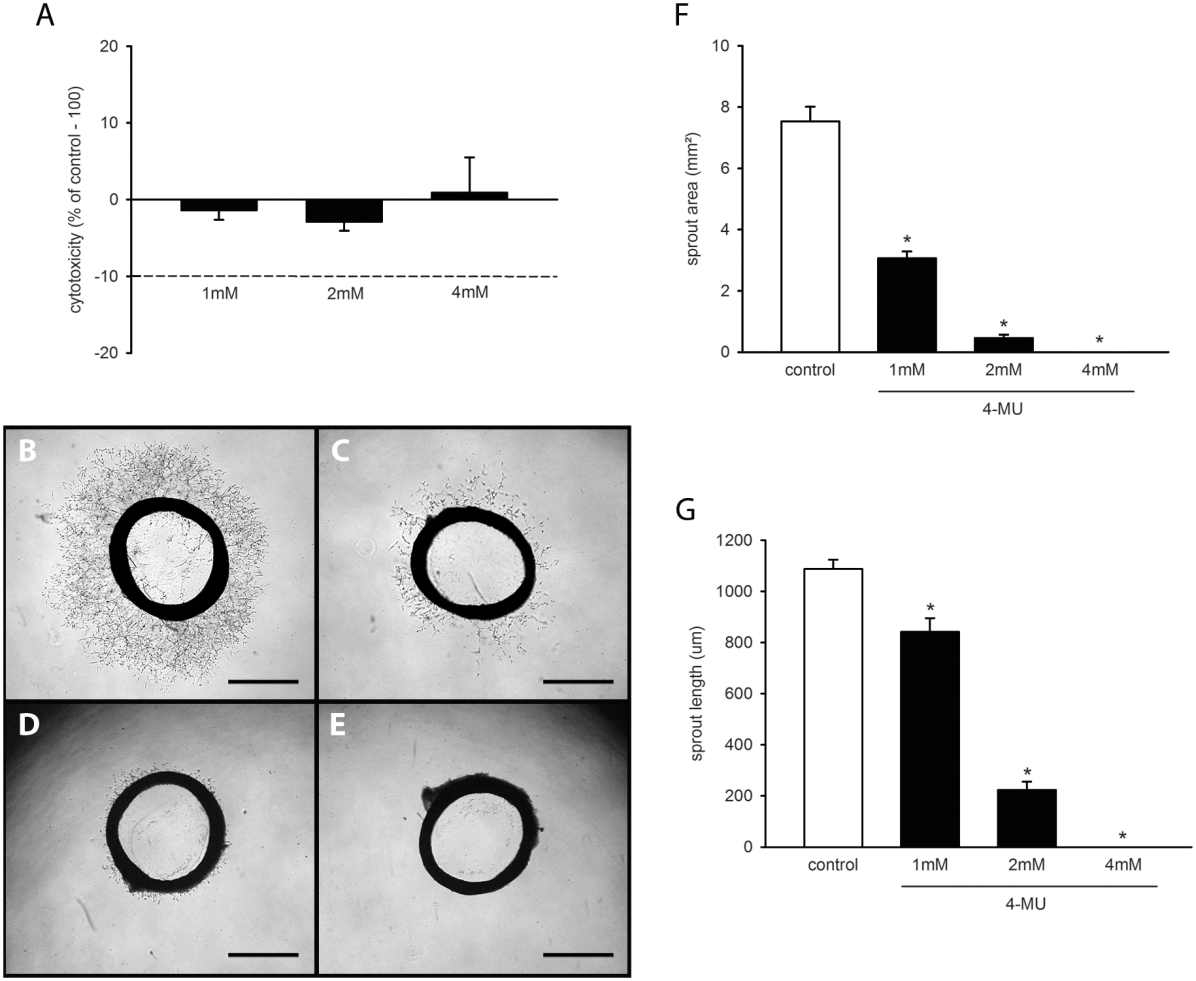
*In vitro* effects of 4-MU. (A) Cytotoxicity (% of control– 100%) of 4-MU on HDMEC, which were exposed for 24h to different doses (1, 2 and 4mM; n = 4 replicates) of 4-MU or vehicle (control; n = 4 replicates), as assessed by LDH assay. Means ± SEM. (B-E) Phase-contrast microscopic images of rat aortic rings with vascular sprouting upon 6 days of treatment with vehicle (B) or 1mM (C), 2mM (D) and 4mM 4-MU (E). Scale bar: 1mm. (F, G) Sprout area (mm^2^) and maximal sprout length (μm) of aortic rings, as assessed by phase-contrast microscopy and computer-assisted image analysis. The aortic rings were exposed to vehicle (control; n = 10 replicates) or increasing concentrations of 4-MU (n = 10 replicates) for 6 days. Means ± SEM. *p<0.05 vs. control.

We first tested the cytotoxicity of 1, 2 and 4mM 4-MU by means of a LDH assay. We found that none of these concentrations induced a relevant release of LDH from HDMEC into the culture medium (Fig 1A). Accordingly, we decided to use these non-cytotoxic concentrations to analyze in a next step the anti-angiogenic activity of the compound in a rat aortic ring assay.

### 4-MU effects on vascular sprouting

In line with previous reports [32,33], cultivation of vehicle-treated aortic rings stimulated the growth of vascular sprouts out of the vessel wall into the surrounding Matrigel. These sprouts finally interconnected with each other to form dense networks of tubular vessel-like structures (Fig 1B). Treatment with 4-MU dose-dependently suppressed this vascular sprouting, as indicated by a significantly reduced sprout area and maximal sprout length when compared to vehicle-treated controls (Fig 1C–1G). Of interest, aortic rings exposed to 4mM 4-MU even exhibited a complete lack of vascular sprout formation during the observation period of 6 days (Fig 1E–1G). These findings demonstrate that non-cytotoxic doses of 4-MU effectively inhibit the formation of new microvessels.

### 4-MU effects on angiogenesis and growth of developing endometriotic lesions

Based on our promising in vitro results, we next analyzed the anti-angiogenic action of 4-MU on developing endometriotic lesions in the dorsal skinfold chamber model by means of intravital fluorescence microscopy [27]. For this purpose, the recipient mice of endometrial fragments were either treated with a daily dose of 20mg/kg or 80mg/kg of 4-MU, because these doses or higher doses have previously been shown to be non-toxic under in vivo conditions [21,23,24]. Accordingly, the animals tolerated the daily treatment with 4-MU well and did not exhibit any differences in activity, feeding and sleeping habits when compared to vehicle-treated controls. The monitoring of body weight revealed that the mice of all three groups lost weight over time (d0: 24.7 ± 1.0g (vehicle), 24.8 ± 0.8g (20mg/kg 4-MU) and 23.0 ± 0.3g (80mg/kg 4-MU) versus d14: 22.2 ± 0.9g (vehicle), 22.1 ± 0.3g (20mg/kg 4-MU) and 21.0 ± 0.3g (80mg/kg 4-MU)). This can be attributed to post-surgical weight loss after chamber implantation and repetitive anesthesia for intravital fluorescence microscopy throughout the observation period of 14 days. However, the body weight and the change of body weight over the 14-day period did not significantly differ between the groups, additionally indicating that treatment with 4-MU did not affect the well-being of the animals.

**Fig 2:**
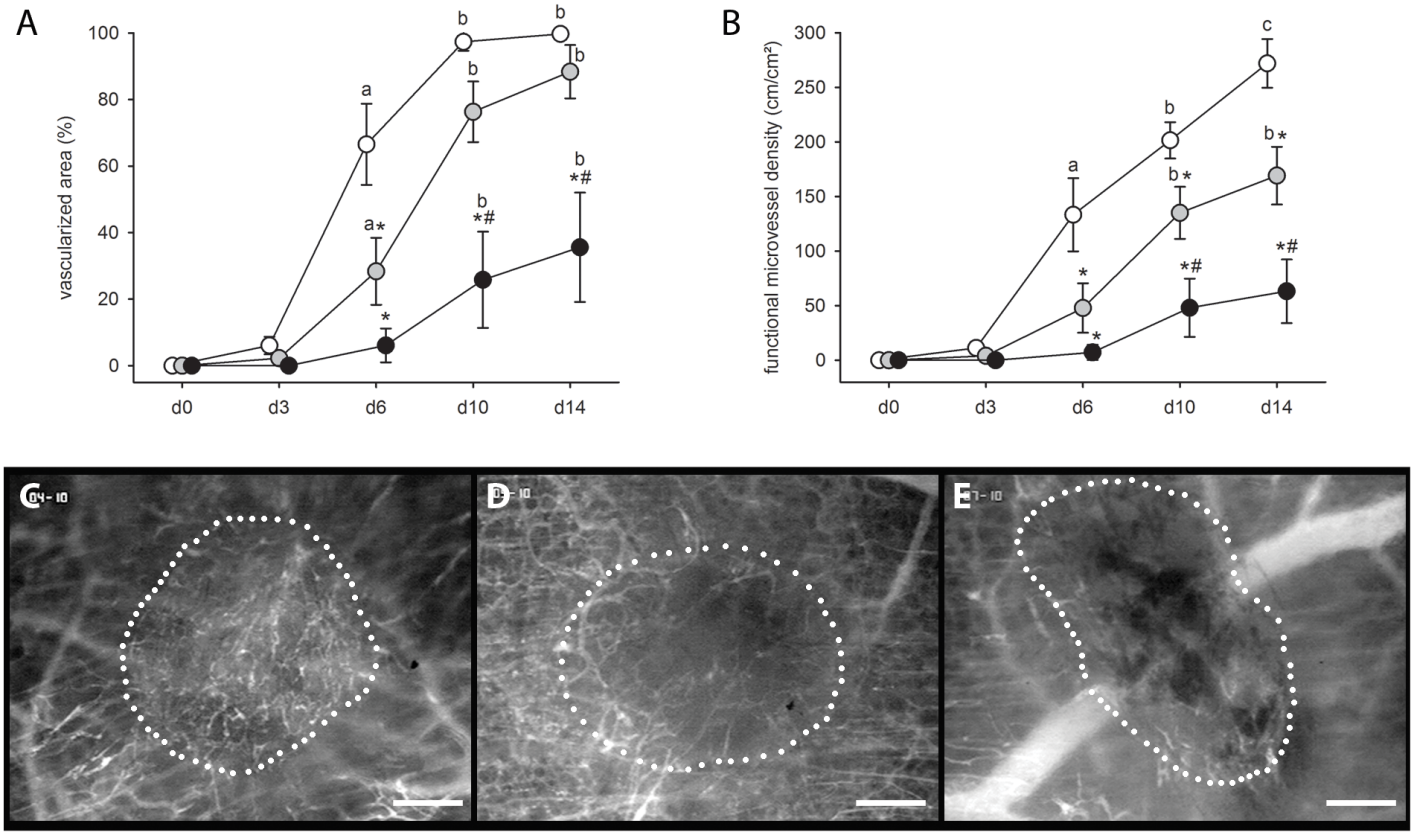
Vascularization of endometriotic lesions in the skinold chamber. (A, B) Vascularized area (%) (A) and functional microvessel density (cm/cm^2^) (B) of developing endometriotic lesions in dorsal skinfold chambers of vehicle-treated controls (white circles; n = 6) and 4-MU-treated (20mg/kg: grey circles, n = 8; 80mg/kg: black circles, n = 7) BALB/c mice, as assessed by intravital fluorescence microscopy and computer-assisted image analysis. Means ± SEM. *p<0.05 vs. control; ^#^p<0.05 vs. 20mg/kg 4-MU; ^a^p<0.05 vs. d0 and d3; ^b^p<0.05 vs. d0, d3 and d6; ^c^p<0.05 vs. d0, d3, d6 and d10. (C-E) Intravital fluorescent microscopic images of endometriotic lesions (borders marked by dotted line) at day 10 after transplantation of endometrial fragments into the dorsal skinfold chamber of a vehicle-treated control (C), a 20mg/kg 4-MU-treated (D) and an 80mg/kg 4-MU-treated (E) BALB/c mouse. Blue light epi-illumination with contrast enhancement by 5% FITC-labeled dextran 150,000 i.v. Scale bar: 200μm.

The transplantation of endometrial fragments onto the striated muscle tissue induced angiogenesis in the dorsal skinfold chamber, resulting in the ingrowth of newly developing vascular sprouts into the grafts of all three groups. However, in line with our in vitro results, this process was dose-dependently suppressed in 4-MU-treated mice. Developing endometriotic lesions in these animals presented with a markedly reduced vascularized area and functional microvessel density between days 6 and 14 when compared to those of vehicle-treated controls (Fig 2A–2E).

**Fig 3:**
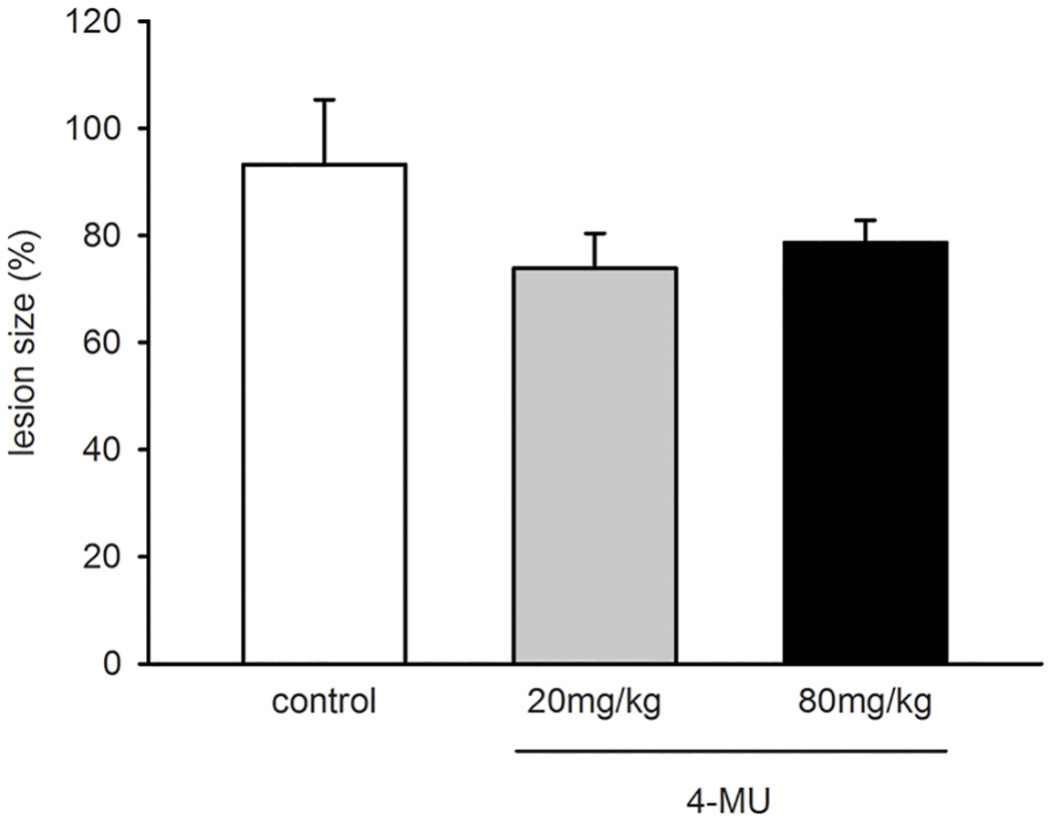
Growth of endometriotic lesions. Lesion size (%) of endometriotic lesions at day 14 after transplantation of endometrial fragments into the dorsal skinfold chambers of vehicle-treated controls (white bar; n = 6) and 4-MU-treated (20mg/kg: grey bar, n = 8; 80mg/kg: black bar, n = 7) BALB/c mice, as assessed by intravital fluorescence microscopy and computer-assisted image analysis. Means ± SEM. p>0.05.

Finally, we assessed the size of endometriotic lesions at day 14 by measuring planimetrically the lesion area on the microscopic images (given in percentage of the area of the endometrial grafts directly after transplantation). Endometriotic lesions in 4-MU-treated animals showed a tendency towards smaller lesion sizes (Fig 3). However, this result was not proven to be significant.

### 4-MU effects on HA synthesis, cell proliferation, apoptosis and microvessel density of established endometriotic lesions

**Fig 4:**
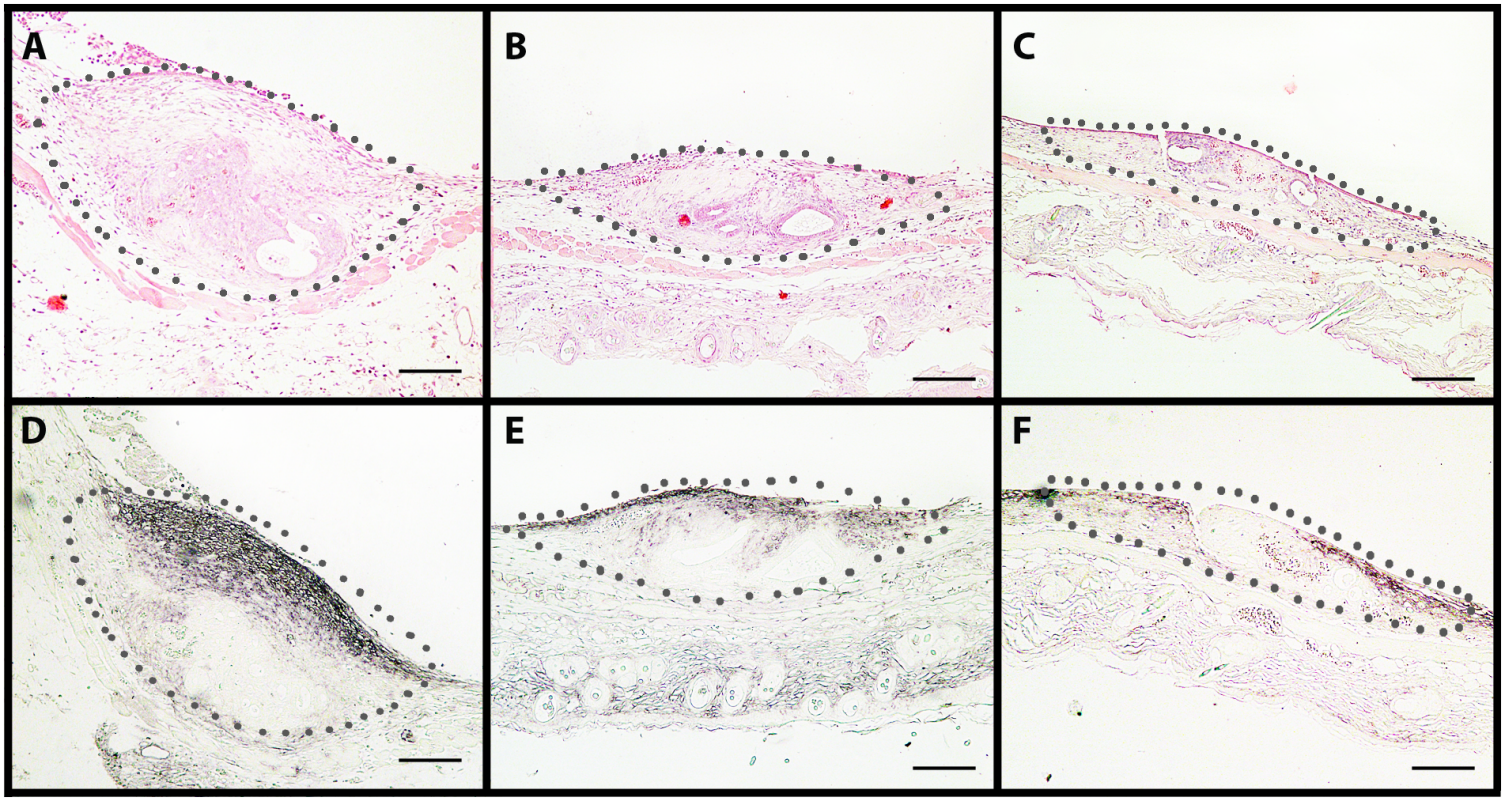
HE and HA stained sections of endometriotic lesions. (A-C) HE-stained cross sections of endometriotic lesions (borders marked by dotted line) at day 14 after transplantation of endometrial fragments into the dorsal skinfold chamber of a vehicle-treated control (A), a 20mg/kg 4-MU-treated (B) and an 80mg/kg 4-MU-treated (C) BALB/c mouse. The lesions show a typical histomorphology with endometrial glands surrounded by stromal tissue. (D-F) Immunohistochemical detection of HA within endometriotic lesions (borders marked by dotted line) at day 14 after transplantation of endometrial fragments into the dorsal skinfold chamber of a vehicle-treated control (D), a 20mg/kg 4-MU-treated (E) and an 80mg/kg 4-MU-treated (F) BALB/c mouse. Scale bar: 100μm. Note the reduced HA expression in the 4-MU-treated lesions (E, F).

At the end of the in vivo experiments, the dorsal skinfold chamber preparations were additionally analyzed by means of histology and immunohistochemistry. HE-stainings revealed that in all three groups the transplanted endometrial fragments had developed to lesions with a histomorphology typical for endometriosis. The lesions contained cyst-like dilated endometrial glands, which were surrounded by a vascularized endometrial stroma (Fig 4A–4C). Semiquantitative analyses of sections corresponding to lesions and surrounding tissue, showed less HA staining of both groups of 4-MU-treated animals (20mg/kg: score 0.8±0.3; 80mg/kg: score 0.8±0.4) when compared to vehicle-treated controls (score 2.3±0.3) (Fig 4D–4F). This indicates that the applied in vivo doses of 4-MU were sufficient to inhibit HA synthesis in the present experimental setting.

**Fig 5:**
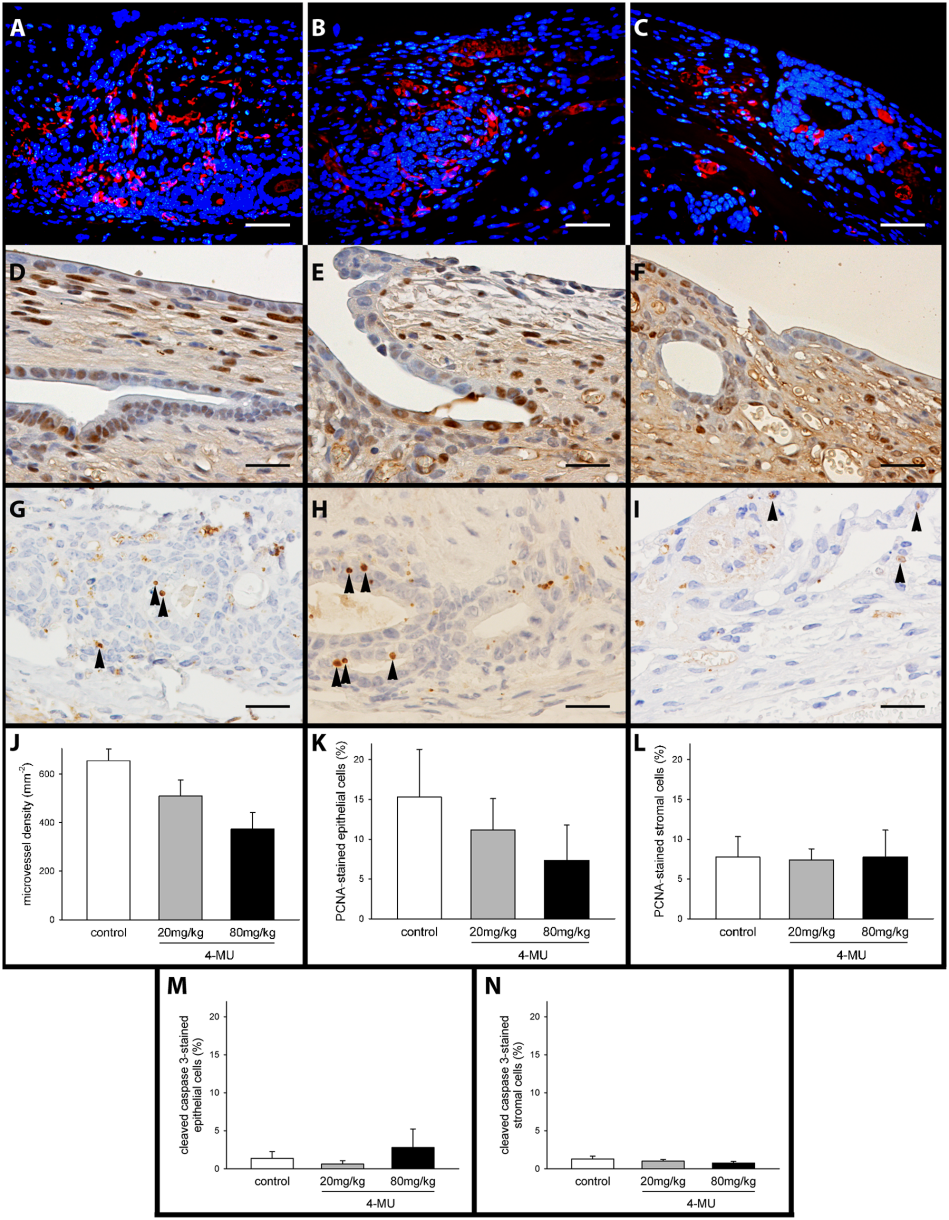
Immunoflluorescent and immunohistochemical detection of markers in sections of endometriotic lesions. (A-C) Immunofluorescent detection of microvessels within endometriotic lesions at day 14 after transplantation of endometrial fragments into the dorsal skinfold chamber of a vehicle-treated control (A), a 20mg/kg 4-MU-treated (B) and an 80mg/kg 4-MU-treated (C) BALB/c mouse. Sections were stained with Hoechst 33342 to identify cell nuclei (blue) and an antibody against CD31 (red) for the detection of microvessels. Scale bar: 25μm. (D-I) Immunohistochemical detection of proliferating PCNA-positive cells (D-F) and apoptotic cleaved caspase-3-positive cells (G-I, black arrow heads) within endometriotic lesions at day 14 after transplantation of endometrial fragments into the dorsal skinfold chamber of a vehicle-treated control (D, G), a 20mg/kg 4-MU-treated (E, H) and an 80mg/kg 4-MU-treated (F, I) BALB/c mouse. Scale bar: 25μm. (J-N) Microvessel density (J, mm^-2^), PCNA-positive (K, L, %) and cleaved caspase-3-positive (M, N, %) epithelial and stromal cells within endometriotic lesions at day 14 after transplantation of endometrial fragments into the dorsal skinfold chambers of vehicle-treated controls (white bar; n = 6) and 4-MU-treated (20mg/kg: grey bar, n = 8; 80mg/kg: black bar, n = 7) BALB/c mice, as assessed by quantitative analysis of immunohistochemical sections. Means ± SEM. *p<0.05 vs. control.

In line with our intravital microscopic results we further detected a dose-dependent reduction of the density of CD31-positive microvessels in 4-MU-treated endometriotic lesions (Fig 5A–5C and 5J). In addition, we performed a correlation analysis between the microvessel density and the HA expression of individual lesions and obtained a Pearson Correlation Coefficient of R = 0.63 (p<0.05), indicating a moderate positive correlation between these two parameters. In contrast, there were no marked differences in the number of proliferating PCNA-positive cells and apoptotic cleaved caspase-3-positive cells in epithelium and stroma within the lesions of all three groups (Fig 5D–5I and 5K–5N).

## Discussion

In recent years, evidence has accumulated that HA plays a key role in the pathogenesis of endometriosis. In the peritoneal cavity, the disease may be initiated by the adherence of menstrual CD44-expressing endometrial cells to mesothelial cells via binding to HA [8,34]. Moreover, CD44-HA interaction also mediates proliferation, inflammation and angiogenesis [35,36], which represent fundamental processes in the formation of new endometriotic lesions and, thus, potential therapeutic targets for the treatment of endometriosis [6,37,38]. Based on these facts, we herein analyzed for the first time the effects of a direct inhibition of HA synthesis in a rodent model of endometriosis.

For this purpose, we used 4-MU, a compound that has been used in various studies for the inhibition of HA synthesis under different experimental conditions [22,39,40]. In vitro, we did not detect any cytotoxic effects on HDMEC of relatively high 4-MU concentrations in the millimolar range. On the other hand, exposure of rat aortic rings to these concentrations effectively suppressed vascular sprout formation, which confirms the results of Garcia-Vilas *et al*. reporting a potent anti-angiogenic activity of the compound [25].

To analyze the effects of 4-MU on developing endometriotic lesions, we used the mouse dorsal skinfold chamber. In contrast to other rodent models of endometriosis, this model allows for the repetitive non-invasive in vivo analysis of angiogenesis in endometriotic lesions by means of intravital fluorescence microscopy [41]. The animals of the present study were daily treated with 20mg/kg or 80mg/kg 4-MU without inducing any alterations in their activity, feeding and sleeping habits. Histological analyses confirmed that these doses are sufficient to inhibit HA synthesis in endometriotic lesions. Our intravital fluorescent microscopic analyses further showed that this treatment dose-dependently reduces the vascularized area and the functional microvessel density of the lesions when compared to vehicle-treated controls.

The inhibition of blood vessel formation by 4-MU has previously been described by Lokeshwar *et al*. [21] in a xenograft prostate cancer model. They treated mice twice daily with 225 or 450mg/kg 4-MU over a period of 35 days without detecting any organ toxicity, changes in serum chemistry or body weight. Our novel results now indicate that much lower doses of 4-MU are already sufficient to suppress angiogenesis in developing endometriotic lesions. Of interest, this was not associated with significant changes in the number of proliferating and apoptotic cells within the lesions when compared to controls. This may be due to the fact that in the present experimental setting the grafted endometrial fragments exhibited a rather small size and tissue thickness. Taking into account that the amount of oxygen, which is usually required for cell survival, can be achieved over a diffusion distance of ~150–200μm from a blood vessel [42,43], major parts of the 4-MU-treated grafts may thus have survived without an own blood supply. Moreover, Svensson et al. [44] reported that inhibition of angiogenesis does not necessarily induce apoptosis and does not suppress tissue proliferation. They treated human neuroblastoma xenografts with the angiogenesis inhibitor TNP-470, which significantly reduced the length and surface area of vessels per tumor without affecting tumor growth or tumor cell apoptosis. Therefore, they concluded that a threshold of angiogenesis inhibition must be reached before significant effects on apoptosis and proliferation occur.

However, these findings may still not completely explain the results of the present study. In fact, previous endometriosis studies performed in the dorsal skinfold chamber model showed the regression of endometriotic lesions treated with rapamycin [45], NS398 (a cyclooxygenase-2 inhibitor) [46], epigallocatechin-3-gallate [47], quinalizarin (a CK2 inhibitor) [29] and telmisartan [31], although the anti-angiogenic efficacy of these compounds was partly lower when compared to 4-MU. This discrepancy may be due to several reasons, including the use of different animal species (hamster vs. mouse) and the relative heterogeneous data distribution in the treatment groups of the present study. Moreover, the compounds may exhibit different pleiotropic activity spectra suppressing proliferation and inducing apoptotic cell death independent of their anti-angiogenic activity. This also applies to 4-MU, which has been shown to suppress important signaling pathways for cell proliferation (Akt, ERK) and to increase markers of the death receptor pathway of apoptosis [21,48,49]. However, our own and the in vitro data of others [25] indicate that 4-MU is capable of inhibiting angiogenesis in much lower concentrations than those needed to affect cell survival. In fact, apoptotic cell death in tumors was induced by application of in vivo doses of up to 450mg/kg 4-MU [21,49]. Therefore, it is possible that the doses used in the present study were not high enough to effectively induce apoptosis.

However, it may also be speculated that the application of 4-MU does not only inhibit angiogenesis but additionally increases ischemic tissue tolerance. For instance, it has been reported that anti-angiogenic treatment of tumors activates signaling pathways such as phosphatidylinositol 3-kinase/Akt, which causes tolerance to hypoxia [50]. A comparable mechanism may have resulted in the survival of endometriotic cells despite a significantly reduced vascularization under treatment with 4-MU. Hence, future studies should indeed clarify whether 4-MU is capable of influencing ischemic tissue tolerance.

Finally, it should be considered in general whether anti-angiogenic compounds, such as 4-MU, are suitable for the establishment of novel strategies for endometriosis therapy. A major challenge in this context is undoubtedly the identification of compounds, which exhibit an acceptable spectrum of side effects without affecting fertility or pregnancy in reproductive-aged endometriosis patients [6]. In addition, an anti-angiogenic endometriosis therapy may only be reasonable in certain stages of the disease, such as during active phases with red endometriotic lesions exhibiting strong angiogenic activity or after surgical treatment to prevent recurrence. In these cases, the combination of anti-angiogenic compounds with well-established drugs may also be suitable to minimize the side effects of the latter by dose reduction and to increase the efficacy of endometriosis treatment. Even though there is still no evidence of this in endometriosis, this possible benefit of anti-angiogenic therapy has already been reported in tumor studies [51].

In summary, the present study demonstrates for the first time that treatment of endometriotic lesions with 4-MU markedly reduces their vascularization. Importantly, this is already achieved in a rather low dose range, which has previously been shown to be well tolerated and not to induce any systemic side effects. Hence, further experimental studies should clarify now whether in the future this anti-angiogenic effect can be used beneficially for the treatment of endometriosis.
